# Acute Cholecystitis-like Presentation in an Adult Patient with Gallbladder Agenesis: Case Report and Literature Review

**DOI:** 10.1155/2020/8883239

**Published:** 2020-12-16

**Authors:** Nahla Elzubeir, Kevin Nguyen, Muhammad Nazim

**Affiliations:** ^1^Department of Internal Medicine, Texas Tech University Health Sciences Center, Amarillo, TX, USA; ^2^School of Pharmacy, Texas Tech University Health Sciences Center, Amarillo, TX, USA; ^3^Department of Surgery, Texas Tech University Health Sciences Center, Amarillo, TX, USA

## Abstract

**Introduction:**

Gallbladder agenesis (GA) is a rare congenital malformation, and majority are asymptomatic; however, symptomatic patients present with a clinical picture similar to biliary colic. Work up usually shows contracted gallbladder (GB) on ultrasound (US), and HIDA scan shows nonvisualization. Magnetic resonance cholangiopancreatography (MRCP) can be helpful in the diagnosis; however, the diagnosis without the latter can only be established intraoperatively. Management should be conservative treatment with antispasmodic drugs. *Case Report*. A 35-year-old female presented to the emergency department with nausea, vomiting, and worsening right upper quadrant (RUQ) abdominal pain. Vitals and laboratory values were unremarkable except for mild leukocytosis, and RUQ US reported “contracted GB, cholelithiasis, 4.2 mm wall thickness, and no ductal dilation.” Surgical consultation was prompted by the diagnosis of acute cholecystitis. The patient was transferred to the operating room for laparoscopic cholecystectomy; however, no GB was found, which was confirmed by intraoperative indocyanine green cholangiography. The procedure was aborted. Postoperatively, CT scan showed absent GB. A HIDA scan showed nonvisualization of the GB after 4 hours. Gastroenterology consultation was suggested to assess for peptic ulcer disease, stricture, or other etiology for her presenting symptoms, and the upper endoscopy showed gastritis. Upper GI with small bowel follow-through study showed mild delayed gastric emptying and contrast in the colon in 45 minutes.

**Conclusion:**

When US imaging findings are equivocal for nonvisualization of GB in a patient with no known history of prior cholecystectomy, additional imaging is required considering the diagnosis of gallbladder agenesis. MRCP is the test of choice. Management is usually conservative with smooth muscle relaxants without the need for surgical operation.

## 1. Introduction

Gallbladder agenesis (GA) is a rare congenital malformation with an incidence of 10–65 per 100,000 [1, 2, 3]. It is more common in females with a 3 : 1 ratio [1]. Most cases are sporadic (around 70%), and there is very little literature on any familial links [1]. Majority of the patients are asymptomatic. However, symptomatic patients present with a clinical picture similar to biliary colic. Work up usually shows a contracted gallbladder on ultrasound, and a HIDA scan shows nonvisualization, which can lead to a false positive interpretation. Magnetic resonance cholangiopancreatography (MRCP) can be helpful in the diagnosis; however, the diagnosis without the latter can only be established intraoperatively. Management should be conservative treatment with antispasmodic drugs.

## 2. Case Report

**Figure 1:**
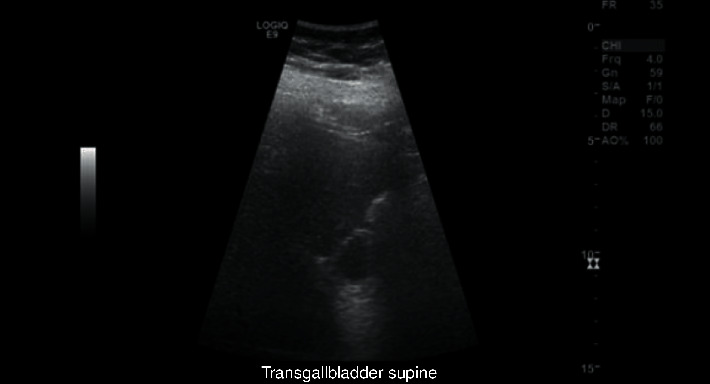
35-Year-old female with biliary colic concerning for acute cholecystitis. Findings: ultrasound image reported as contracted gallbladder.

**Figure 2:**
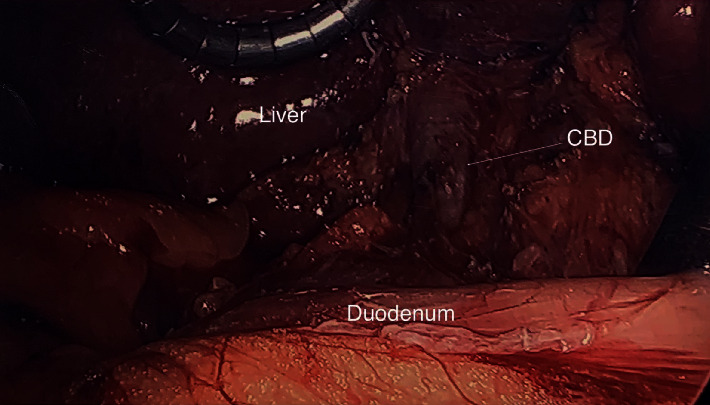
35-Year-old female with biliary colic concerning for acute cholecystitis. Findings: intraoperative photograph showing common bile duct (CBD arrowed), the liver, and the duodenum with the absence of a gallbladder.

**Figure 3:**
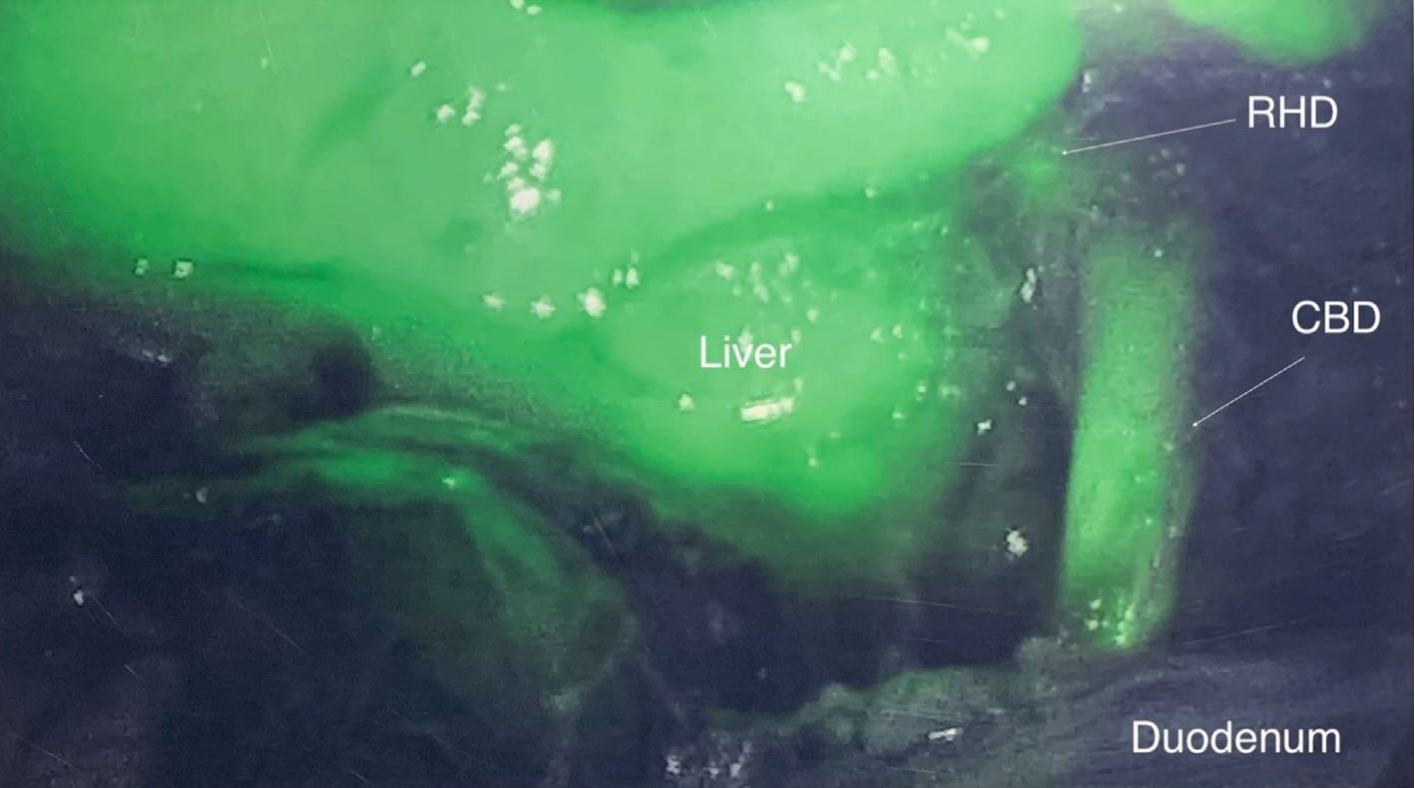
35-Year-old female with biliary colic concerning for acute cholecystitis. Findings: intraoperative photograph showing the use of indocyanine green contrast viewing the common bile duct (CBD arrowed), the right hepatic duct (RHD arrowed), the liver, and the duodenum with the absence of a gallbladder.

**Figure 4:**
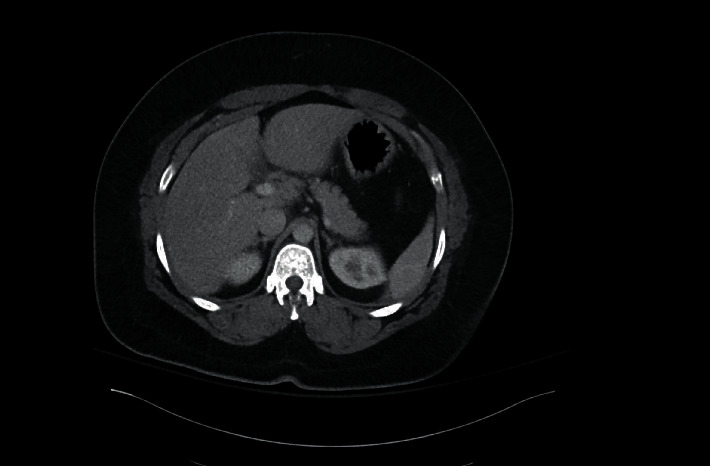
35-Year-old female with biliary colic concerning for acute cholecystitis. Findings: contrast-enhanced CT images of the abdomen in the portal venous phase in the sagittal (1a), coronal (1b), and axial (1c) planes demonstrate empty gallbladder fossa. Technique: axial CT with sagittal and coronal reconstructions, 158 mA, 120 kV, 3 mm slice thickness, and 80 ml Omnipaque 350 intravenous contrast.

**Figure 5:**
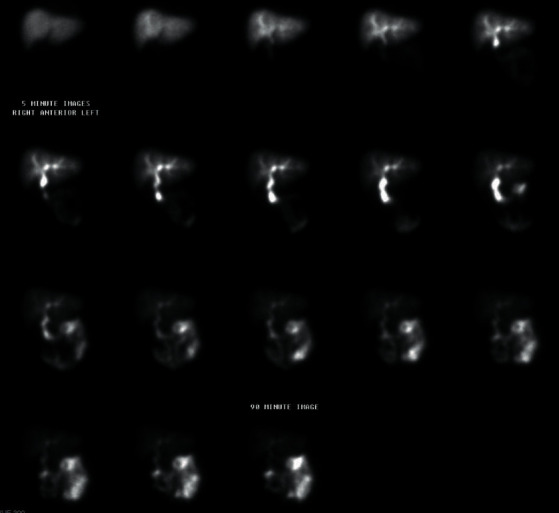
35-Year-old female with biliary colic concerning for acute cholecystitis. Findings: HIDA scan demonstrating nonvisualization of the gallbladder.

We present a 35-year-old obese female with a body mass index (BMI) of 41, with otherwise no significant past medical or surgical history, who presented to the emergency department (ED) with nausea, vomiting, and worsening right upper quadrant (RUQ) abdominal pain for a few days. The pain worsened after meals, and she has had intermittent pain after eating for one year. In the ED, vitals were stable with a BP of 127/78, an HR of 96, an RR of 20, and a tender RUQ on examination. Work up revealed a white blood cell count (WBC) of 12,000 and an unremarkable liver function test (LFT), and RUQ ultrasound reported a “contracted gallbladder with cholelithiasis with gallbladder wall thickness at 4.2 mm. No ductal dilatation seen” (Figure 1). Surgical consultation was consistent with acute cholecystitis, and the patient was taken to the operating room for laparoscopic cholecystectomy. Upon laparoscopic evaluation, no gallbladder was found (Figure 2). This was further confirmed by intraoperative indocyanine green (ICG) dye cholangiography (Figure 3). The procedure was aborted. CT scan was performed postoperatively which showed an absent gallbladder (Figure 4). The patient also underwent a HIDA scan that showed nonvisualization of the gallbladder after 4 hours (Figure 5), suggesting the diagnosis of GA. The gastroenterologist was consulted to assess for peptic ulcer disease, stricture, or other etiology for her presenting symptoms who performed esophagogastroduodenoscopy (EGD) that showed *H. pylori* negative gastritis. Upper GI with small bowel follow-through study along with gastric emptying study were performed. Small bowel follow-through showed contrast in the colon in 45 minutes. Gastric emptying study revealed mild delay.

## 3. Discussion

The first case of GA was described in 1701 by Lemery [1, 4]. GA is a rare congenital malformation with an incidence of 10–65 per 100,000 [1, 2, 3, 6]. It is more common in females with 3 : 1 ratio [1]. Most cases are sporadic (around 70%), and there is very little literature on any familial links [1]. The first literature review of GA was published in 1988 by Bennion et al. [4], who at the time categorized GA into 3 types after reviewing 12 patients at their health system and applied the same principles to an additional 382 patients from the literature. The 3 categories are as follows: (a) patients with multiple fetal anomalies, this group commonly dies of other congenital defects; (b) the asymptomatic group, an incidental finding at autopsy; and (c) the symptomatic group, which usually presents with a clinical picture suggesting biliary disease. The mechanism of GA is attributed likely to a failure of the common bile duct bud to proliferate or canalize to develop into the cystic duct and gallbladder in the 5th gestational week [2]. Our patient falls into the 3rd category, of which 50% of the cases of GA are assigned in this group [4].

The challenge still presents in establishing the diagnosis of GA before the patient undergoes unnecessary operation, whether laparoscopic or with laparotomy. The symptomatic patients typically present with biliary colic-postprandial pain in the abdomen, associated sometimes with nausea and vomiting. The main reason of diagnostic complications is a “constricted, shrunken gallbladder” and sometimes hyperechogenic shadows seen by radiologists under ultrasonography, findings which are misinterpreted and then considered as gallstones. The patient is mistakenly diagnosed with cholelithiasis and later is operated on. These false positive ultrasonography findings have been previously described as possible duodenal wall and gas shadows by Hammond [5]. Our case reiterates this typical mistake, which was described in an almost identical manner by Pipa et al. [6]. A HIDA scan helps to further confirm the false positive diagnosis of acute cholecystitis, “nonvisualization” of the gallbladder, which is in fact, absent. The dilemma remains how to avoid unnecessary surgery, and how GA can be diagnosed before surgery. Malde in 2010 presented an algorithm for the cases which are manifested with similar symptoms as biliary colic [3]. According to this algorithm, it is necessary to perform another radiological investigation, such as MRCP, computed tomography (CT), endoscopic retrograde cholangiopancreatography (ERCP), and endoscopic ultrasound, if it is impossible to visualize the gallbladder under abdominal ultrasound, or if it is seen as shrunk with a description of chronic cholelithiasis [3, 1]. The diagnosis of GA is helped by the above-listed examinations, which could be treated conservatively. From all of these methods, MRCP is the diagnostic test of choice, taking into consideration its informative character in the diagnosis of GA and it being a noninvasive test. We think that the other three methods should be chosen if MRCP could not be performed or contraindicated.

## 4. Conclusion

The diagnosis of GA should be considered in cases where diagnosis is inconclusive from the usual standard of care investigation, ultrasound. When a shrunken/contracted gallbladder is detected on ultrasound, additional radiological examinations are required. MRCP is considered as the diagnostic test of choice. If a HIDA scan was performed, the “nonvisualization” of the gallbladder should further prompt MRCP. If the clinical diagnosis of GA was made, management for asymptomatic patients is usually conservative with smooth muscle relaxants without the need for surgical operation.
